# Assessing alternative strategies to control almond red leaf blotch through the reduction of *Polystigma amygdalinum* inoculum in leaf litter

**DOI:** 10.1002/ps.70192

**Published:** 2025-09-08

**Authors:** Gemma Pons‐Solé, Lidia Aparicio‐Durán, Jordi Luque, Laura Torguet, Xavier Miarnau

**Affiliations:** ^1^ IRTA Sustainable Plant Protection Program, Centre de Cabrils Cabrils Catalonia Spain; ^2^ IRTA Fruit Production Program, Fruitcentre Lleida Catalonia Spain

**Keywords:** almond, cultural practices, disease management, *Polystigma amygdalinum*, *Prunus amygdalus*, red leaf blotch

## Abstract

**Background:**

Red leaf blotch (RLB), caused by *Polystigma amygdalinum*, is a major foliar disease of almond trees in Mediterranean and Middle Eastern regions. While preventive fungicide applications are the main control strategy, cultural practices aimed at reducing pathogen inoculum in leaf litter are gaining relevance. This study evaluated the efficacy of four chemical treatments on fungal biomass and ascospore production in leaf litter and assessed the impact of two cultural practices—urea application and leaf litter removal—on airborne inoculum levels and disease incidence under field conditions.

**Results:**

Urea and lime sulfur significantly reduced ascospore production by 99% and 94%, respectively, compared to the untreated control. Urea was the only treatment that also significantly reduced fungal biomass. In field trials, leaf litter removal reduced airborne ascospores by 60% but did not significantly affect RLB incidence or severity. Urea application reduced airborne ascospores by 45% and led to a modest yet statistically significant 4% reduction in disease incidence.

**Conclusion:**

This study highlights the potential of cultural practices to reduce the primary inoculum of *P. amygdalinum* in almond orchards. Urea application offers dual benefits by reducing both fungal biomass and ascospore release, contributing to modest disease suppression. For more effective and sustainable RLB control, these practices should be integrated into a broader management strategy that includes the selection of tolerant almond cultivars, preventive fungicide applications guided by forecasting models. © 2025 The Author(s). *Pest Management Science* published by John Wiley & Sons Ltd on behalf of Society of Chemical Industry.

## Graphical abstract

Cultural practices offer effective RLB disease management strategies. Urea application or removal of almond leaf litter reduces the primary inoculum of *Polystigma amygdalinum*.

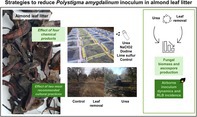

## 1 INTRODUCTION

Almond (*Prunus amygdalus Batsch*) is currently considered the most important nut crop worldwide, with a global production of 3.52 million t of in‐shell almonds in 2023.^1^, ^2^ The leading producer countries over the last decades include the United States and Australia (which respectively accounted for the 50.8% and 7.4% of the 2023 global production), where most of the almond orchards are managed under an intensive irrigated system. In contrast, almonds have traditionally been cultivated under marginal rainfed conditions in the Mediterranean basin and Middle East regions. To enhance the low yields attributed to this traditional approach, the almond industry in these countries is currently facing the challenge of transitioning from traditional rainfed systems to more technical and intensive irrigated systems.^3^, ^4^ Thus, Spain ranked third in world global production (6.8% in 2022), while ranked first in almond growing acreage in the same year, with around 760.000 ha (32.3%).^1^ Nevertheless, factors like irrigation and high tree density, along with the climatic conditions of the new almond growing areas and the adoption of new disease‐susceptible almond cultivars, have been associated with the rise in incidence and prevalence of various new and reemerging almond diseases such as anthracnose, Diaporthe dieback and twig canker and red leaf blotch.^5^, ^6^, ^7^

Red leaf blotch (RLB), caused by the ascomycete *Polystigma amygdalinum*, is currently one of the most prevalent and concerning foliar diseases affecting almond crop in the Mediterranean and Middle East regions,^8^, ^9^ and the disease has been recently detected in California (USA).^10^ The pathogen overwinters in almond leaf litter, where perithecia develop and give rise to the production of ascospores. In spring, under favorable temperature and humidity conditions, mature ascospores are released and dispersed through the air to infect new almond leaves.^11^, ^12^ Following infection, RLB symptoms appear after an incubation period that can extend up to 10 weeks.^11^, ^13^ Initial symptoms are manifested as yellowish spots on both sides of the leaf, evolving into orange‐reddish lesions, which sizes expand over time, with the formation of the fungal stromata on leaves. As a result, an early defoliation of trees can be observed, therefore diminishing tree photosynthetic activity and potentially affecting crop yield.^8^, ^14^ RLB is considered a monocyclic disease, in which the only inoculum source are the ascospores formed in perithecia in fallen leaves infected in the preceding year.^15^, ^16^

Although differential levels of RLB susceptibility among almond cultivars have been reported^5^, ^17^, ^18^, ^19^; no cultivars have been identified to date as entirely resistant to RLB. Consequently, RLB control currently relies extensively on protecting almond trees through preventive fungicide applications. In this sense, several studies have assessed the efficacy of various fungicides for RLB control.^11^, ^20^, ^21^ With the objective of optimizing the seasonal fungicide application program, by reducing the number of fungicides applications without significantly increasing RLB incidence, Pons‐Solé *et al*.^16^ recently developed an epidemiological model to predict the amount of *P. amygdalinum* airborne ascospores in NE Spain based on meteorological variables. The model's performance was assessed to optimize RLB fungicide programs, achieving approximately a 50% reduction in fungicide applications, with only a slight (3%) increase in RLB incidence compared to a standard calendar‐based fungicide program.

Additionally, some cultural practices have been recommended to reduce the inoculum source.^8^, ^22^, ^23^, ^24^ These practices are applied to leaf litter and involve: (i) burning, burying or physical removal of leaf litter, and (ii) its treatment with crystalline urea to accelerate its decomposition. However, their efficacy to improve RLB control has been rarely demonstrated in practice. Recently, López‐Moral *et al*.^24^ explored the use of two saprophytic microorganisms (*Myrothecium inundatum* and *Fusarium oxysporum*) as a biological alternative to reduce *P. amygdalinum* inoculum in almond leaf litter in the framework of organic agriculture. They found that *M. inundatum* significantly reduced the number of ascospores and the development of perithecia in leaf litter, showing a similar effect to that of urea treatments.

Research on the efficacy of the cultural practices to reduce *P. amygdalinum* inoculum has primarily focused on assessing ascospore presence in leaf litter but has not considered the actual airborne dispersal of ascospores and subsequent infection levels on orchards. Moreover, while these cultural practices have been assumed as essential parts of RLB control strategies, the direct impact of such practices on disease incidence has never been evaluated, to the best of our knowledge. Expanding knowledge on the effectiveness of these cultural practices, along with optimizing their application, could contribute to reducing fungicide use within integrated production systems. Therefore, the main objectives of this study were: (i) to evaluate the effect of different chemical products on the *P. amygdalinum* inoculum biomass and ascospore production in leaf litter, and (ii) to assess the effect of the two most recommended cultural practices (i.e., urea treatment and leaf litter removal) on the airborne inoculum dynamics and the subsequent RLB incidence and severity under natural field conditions.

## 2 MATERIALS AND METHODS

### 2.1 Experimental orchards

Experiments were conducted over the period from 2020 to 2023 in two almond orchards located in NE Spain, where RLB occurs naturally.^19^ The first orchard was located in les Borges Blanques, Lleida, Spain (Borges hereafter; UTM coordinates: WGS84 Datum 31 T *X* = 320 870, *Y* = 4 597 530). This orchard is an experimental plot owned by IRTA which was planted in 2009 with 21 cultivars grafted onto ‘INRA GF 677’ rootstock, pruned as central axis, and with a tree spacing of 4 × 2 m. The second orchard was located in Vilagrassa, Lleida, Spain (*X* = 341 313, *Y* = 4 612 125). This orchard is a commercial plot of ‘Tarraco’ cultivar grafted onto ‘INRA GF 677’ rootstock, planted in 2007, pruned as open‐vase, and with a tree spacing of 7 × 6 m. Both orchards were drip irrigated and managed according to the Spanish Integrated Production Management practices.^25^ No fungicide treatments were applied throughout the experimental period in either orchard.

### 2.2 Evaluation of chemical products to control *P. amygdalinum* on leaf litter

In December 2021, RLB symptomatic fallen leaves from different varieties were collected at Borges orchard, and groups of 60 leaves were enclosed in plastic mesh bags measuring 30 × 40 cm. Bags were individually labelled and separately treated with either one of four chemical products: crystalline urea (trade name: Krystafeed [Tarazona Agrosolutions, Silla, Spain]; active ingredient content: 46%; dose: 100 g L^−1^), dodine (Syllit [UPL Iberia, Barcelona, Spain]; a.i. content: 54.4%; dose: 3 g L^−1^), chlorine dioxide precursor (Somagro [Somvital SL, Zaragoza, Spain]; a.i. content: 40% NaHSO_4_ + 24% NaClO_2_; dose: 0.06 g L^−1^; referred as NaClO_2_ hereafter), and lime‐sulfur (Curatio [Andermatt Iberia, Valladolid, Spain]; a.i. content: 38%; dose: 125 g L^−1^). Additionally, a control group was included, by treating the bags with tap water instead of any chemical product. All products were gently applied (i.e., until run‐off) using a manual sprayer.

Bags were placed on the ground in the Borges orchard and periodically collected on three different times in 2022 (February 2 March 2, and May 2; hereafter referred to as t1, t2, and t3, respectively). A total of 60 bags were prepared, representing the five treatments and the three different collecting dates, with four replicates per combination. The experiment was repeated in 2023 growing season following similar procedures, but with only one sampling time on May 9, 2023 (t3). Thus, only 20 bags were used in this repetition. We decided to perform a single repetition in 2023 after preliminary analyses of 2022 data, which confirmed that the best date to find consistent statistical differences between leaf litter treatments was the third sampling time (t3).

In both experiment repetitions, bags were collected, taken to the laboratory, and leaves were dried in oven for 48 h at 35 °C and partially ground in a mortar. Subsequently, two subsamples of approximately 1 g (dry weight) were finely ground in a mortar with 40 mL of sterilized deionized water for about 15 min. The resulting homogenized suspensions were filtered through a two‐folded Nylon mesh and used for further analyses.

The presence of *P. amygdalinum* in sample suspensions was quantified by two different methods: (i) ascospore counts, and (ii) fungal biomass estimation using real‐time quantitative PCR (qPCR). Ascospores were identified under a microscope (×250) based on their morphological characteristics and counted using a hemocytometer (Neubauer chamber). Four independent counts were performed for each subsample, with eight counting replicates per counting. Ascospore concentration was expressed as the estimated number of ascospores per gram of dry leaf weight (asc g^−1^, hereafter). Subsequently, DNA was extracted from 0.5 mL of the leaf homogenate suspensions using the E.Z.N.A.® Plant DNA Kit (Omega Bio‐Tek, Norcross, GA, USA). Fungal biomass of *P. amygdalinum* was estimated through qPCR, using the species‐specific primer pair PamyI2F4/PamyI2R2 designed by Zúñiga *et al*.^21^ The qPCR was conducted in a StepOnePlus thermal cycler (Life Technologies, Carlsbad, CA, USA), with three technical replicates for each DNA sample starting from 1.0 to 1.1 g leaf dry weight. Fungal biomass was indirectly expressed as the quantification cycle value (Cq)^26^, ^27^ since available standard curves prepared from known ascospore suspensions in our lab never included those lower Cq values observed when processing leaf homogenate suspensions, and hence, the impossibility to accurately estimate the fungal biomass.

### 2.3 Evaluation of cultural practices on airborne ascospores dynamics and RLB incidence

In 2020 and 2022, three Hirst 7‐day recording volumetric spore traps (Burkard Manufacturing Co. Ltd., Rickmansworth, Hertfordshire, UK) were placed in the Vilagrassa orchard. Each trap was centrally positioned within a plot corresponding to the following treatments: physical removal of the leaf litter, application of crystalline urea (Krystafeed; dose: 25 g L^−1^) on the leaf litter, and an untreated control. Treatments were implemented during the preceding autumn (November–December) for both evaluated years. Each treatment was applied to a plot of approximately 1.6 ha with about 390 trees.

Spore traps operated continuously from weeks 15 (early April) to 23 (early‐June), as it was known that most *P. amygdalinum* ascospores are released during this period in the region.^12^, ^16^ The intake orifice of the volumetric trap was at 0.45 m above the ground and the air flow was set at 10 L min^−1^. Airborne particles were captured on a Melinex® 200 gauge (TEKRA, New Berlin, WI, USA) transparent plastic tape coated with an adhesive silicone solution (Lanzoni, Bologna, Italy) and rotating at 2 mm h^−1^. Plastic tapes were collected and replaced weekly, and DNA was extracted from daily tape fragments using the E.Z.N.A.® Plant DNA Kit as described by Zúñiga *et al*.^28^ Daily amounts of *P. amygdalinum* ascospores were estimated through qPCR following procedures as described above. Finally, daily concentrations of *P. amygdalinum* airborne ascospores were expressed as ascospores m^−3^ day^−1^ (asc m^−3^ day^−1^) and summarized as weekly cumulative concentrations.

For both experimental years, RLB symptoms were evaluated in late July (2020) or early August (2022) on nine ‘Tarraco’ almond trees adjacent to each spore trap. From each tree, 30 leaves were arbitrarily selected from new shoots, considering different orientations and heights. RLB incidence and severity were visually assessed following the procedures described by Miarnau *et al*.^19^ Leaves were categorized based on the estimated percentage of affected leaf surface into the following classes: class 0 (0% affected leaf surface), 1 (1%–10%), 2 (11%–20%), 3 (21%–50%), and 4 (>50%). RLB incidence was calculated as the proportion of leaves exhibiting at least one RLB lesion, whereas RLB severity was calculated from the mean percentage of affected leaf surface.

### 2.4 Statistical analysis

All experiments were repeated in different almond growing seasons to get consistent results. In all statistical analyses, the repetition of experiments was considered as a random factor, whereas all other factors (e.g., leaf management practices in the field and chemical treatments on leaves) were fixed. In the experiment on the efficacy of different chemicals, independent analyses were done for each sampling time (t1, t2, and t3) since the objective of this preliminary trial was to define the best period to show effective differences among treatments in terms of ascospore production and fungal biomass in leaves. In each individual experiment, a completely randomized design was used to allocate the experimental units. All statistical analyses were performed using R software version 4.3.1.^29^

Disease incidence data were analyzed using a generalized linear modeling (GLM) approach by considering the frequencies of RLB symptomatic and asymptomatic leaves and, specifically, using an error distribution from the binomial family. We therefore used the *glmer* function of the *lme4* package.^30^ Disease severity values were analyzed as proportions. In this case and the remaining dependent variables and analyses, a linear modeling approach was used by using the function *lm* included in the *stats* package. Prior to analyses, ANOVA assumptions were checked with the *gvlma* function of the *gvlma* package.^31^, ^32^ When necessary, data were transformed to meet the ANOVA assumptions, with *arcsin* transformation before the analyses. After modeling, mean comparisons among treatments were performed using the package *emmeans*,^33^ and Tukey–Kramer's *post hoc* test was set as the method for comparisons. Significance level was declared at *α* = 0.05 in all analyses.

## 3 RESULTS

### 3.1 Evaluation of chemical products to control *P. amygdalinum* on leaf litter

**Figure 1:**
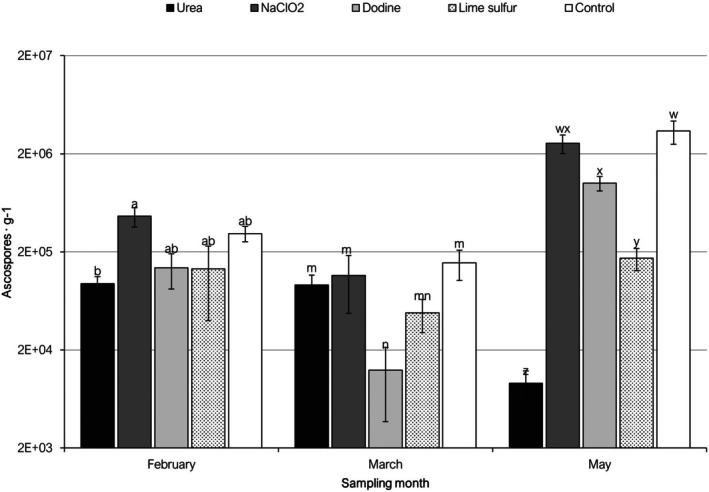
Mean amounts of *Polystigma amygdalinum* ascospores in almond leaf litter, expressed as ascospores per gram of dried leaf weight (ascospores·g^−1^), treated with different chemical products and collected at different sampling dates in 2022. Different letters, in each sampling date, indicate significant differences according to Tukey–Kramer's test (*P* < 0.05). The error bars indicate the standard error of the mean.

**Figure 2:**
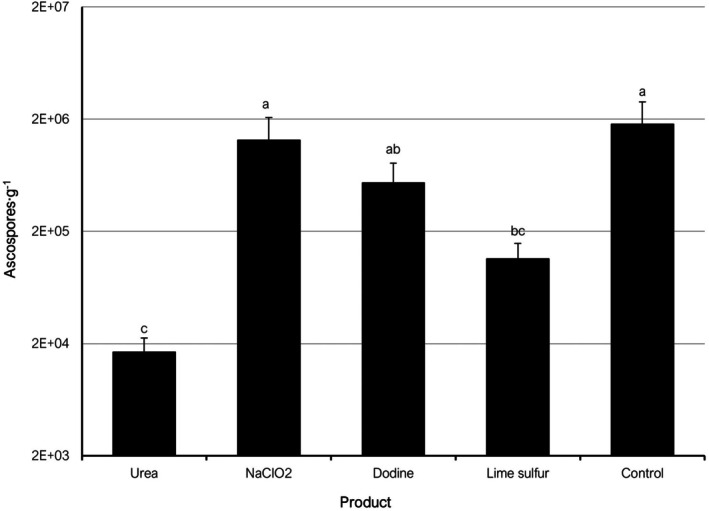
Mean amounts of *Polystigma amygdalinum* ascospores in almond leaf litter, expressed as ascospores per gram of dried leaf weight (ascospores·g^−1^), treated with different chemical products and collected in May of 2022 and 2023 seasons (combined data). Different letters indicate significant differences according to Tukey–Kramer's test (*P* < 0.05). The error bars indicate the standard error of the mean.

Microscopic observations revealed the presence of ascospores of *P. amygdalinum* in all leaf litter samples treated with different chemical products in 2022 and 2023, with different concentrations depending on the sampling time and treatment (Figs. 1 and 2). In 2022, at the initial sampling time t1 (February), mean concentration values ranged between 95 051 asc g^−1^ in the urea treatment and 461 367 asc g^−1^ in the NaClO_2_ treatment. However, no significant differences were observed between the different treatments and the untreated control. At sampling time t2 (March), mean values ranged from 12 448 asc g^−1^ in the dodine treatment to 154 649 asc g^−1^ in the untreated control. Significant differences in mean ascospore amounts were observed between the dodine treatment, showing the lowest value, compared to all other treatments, except for lime‐sulfur. At sampling time t3 (May), mean values ranged between 9145 asc g^−1^ in the urea treatment and 3 409 314 asc g^−1^ in the untreated control. For this sampling time, all evaluated products but NaClO_2_ resulted in significantly lower ascospore concentrations than in the control treatment. In addition, the ascospore amount in the urea treatment was the lowest for all treatments and sampling dates (Fig. 1). Overall, the highest ascospore concentrations in the untreated control were detected in the t3 sampling time as compared to previous sampling dates. A similar pattern was observed for all other treatments excluding urea, which showed the lowest ascospore amount in t3 than in previous dates (Fig. 1).

In 2023, a single sampling time was performed at t3 (May) that resulted in mean values of ascospore amounts ranging between 24 557 asc g^−1^ in the urea treatment and 205 562 asc g^−1^ in the untreated control. In general, ascospore amounts in 2023 were lower than in 2022 for all products except urea (Fig. S1). Specifically for the sampling time t3 in 2023, all evaluated products except dodine resulted in significantly lower ascospore concentrations than in the control (Fig. S1). When analyzing the combined data of the t3 sampling corresponding to 2022 and 2023, only urea and lime‐sulfur significantly reduced ascospore concentrations as compared to the control (Fig. 2).

**Figure 3:**
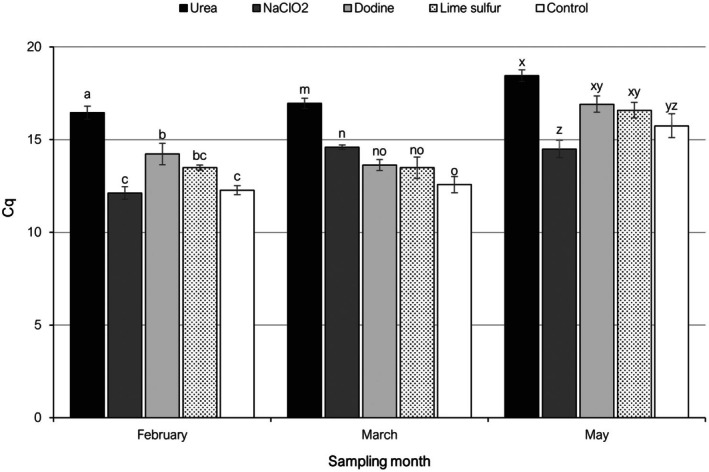
Mean quantification cycle (Cq) values from qPCR analyses targeting *Polystigma amygdalinum* in almond leaf litter treated with different chemical products and collected at different sampling dates in 2022. Different letters, in each sampling date, indicate significant differences according to Tukey–Kramer's test (*P* < 0.05). The error bars indicate the standard error of the mean.

The estimations of *P. amygdalinum* biomass through qPCR among the different leaf treatments evaluated in 2022 are shown in Fig. 3. At sampling time t1, mean Cq values ranged between 12.12 (NaClO_2_) and 16.46 (urea). Only dodine and urea treatments resulted in significantly higher Cq values than in the untreated control. At t2, mean Cq values ranged between 12.58 (control) and 16.96 (urea). Urea and NaClO_2_ treatments showed significantly higher Cq values than in control. At t3, mean Cq values ranged between 14.49 (NaClO_2_) and 18.45 (urea). However, only the urea treatment led to a significantly higher Cq value as compared to the control (Fig. 3).

**Figure 4:**
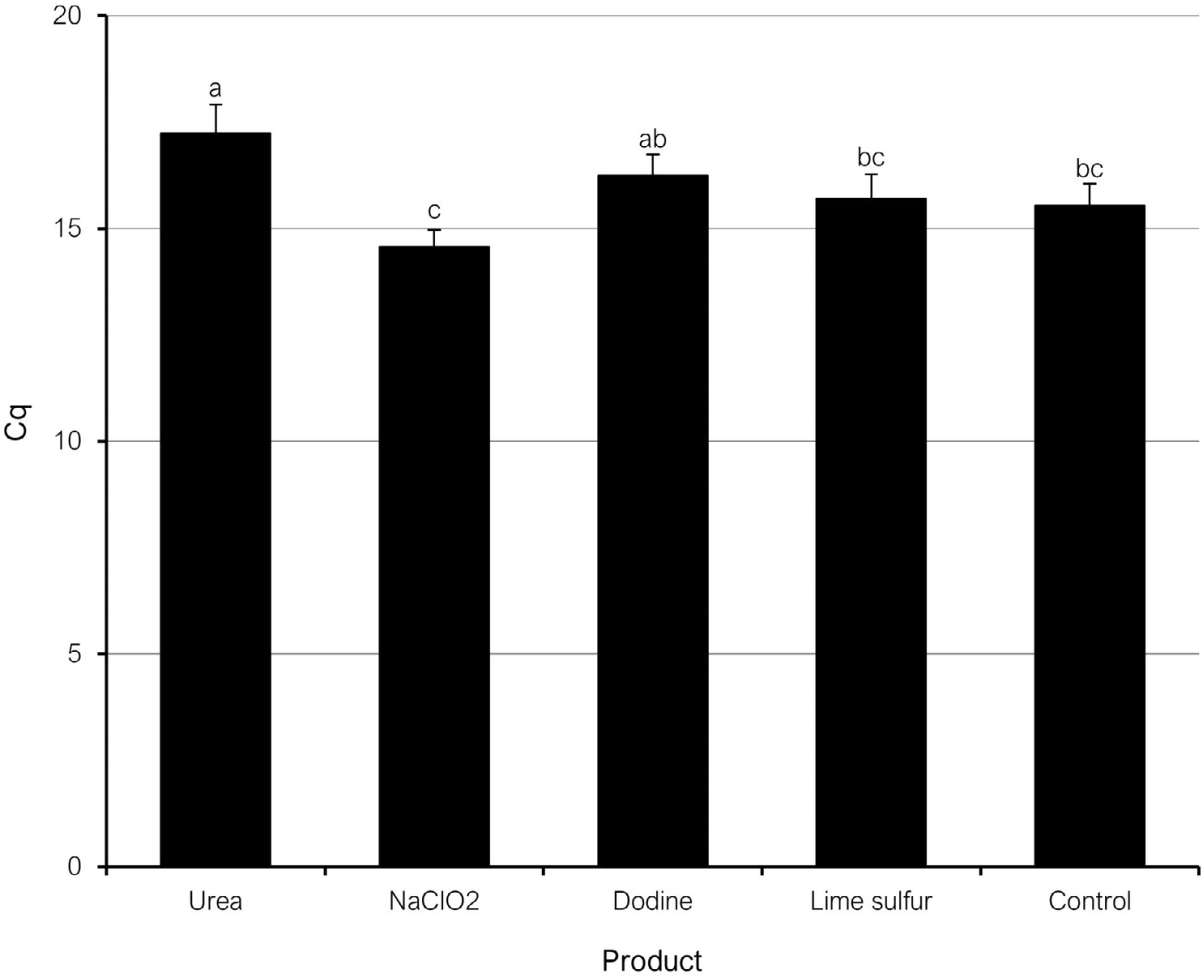
Mean quantification cycle values (Cq) from qPCR analyses targeting *Polystigma amygdalinum* in almond leaf litter treated with different chemical products and collected in May of 2022 and 2023 seasons (combined data). Different letters indicate significant differences according to Tukey–Kramer's test (*P* < 0.05). The error bars indicate the standard error of the mean.

In 2023, mean Cq values measured at sampling time t3 ranged between 14.65 (NaClO_2_) and 16.01 (urea), as similarly occurred in 2022 (Fig. S2). However, no treatments showed mean Cq values significantly different from the untreated control. When analyzing the combined dataset of the t3 samplings corresponding to 2022 and 2023, urea was the only product that showed significantly higher Cq values than the control (Fig. 4). Hence, in the assessment of *P. amygdalinum* biomass by qPCR, urea was the only product able to significantly reduce the fungal biomass in treated leaves.

### 3.2 Evaluation of cultural practices on airborne ascospores dynamics and RLB incidence

**Figure 5:**
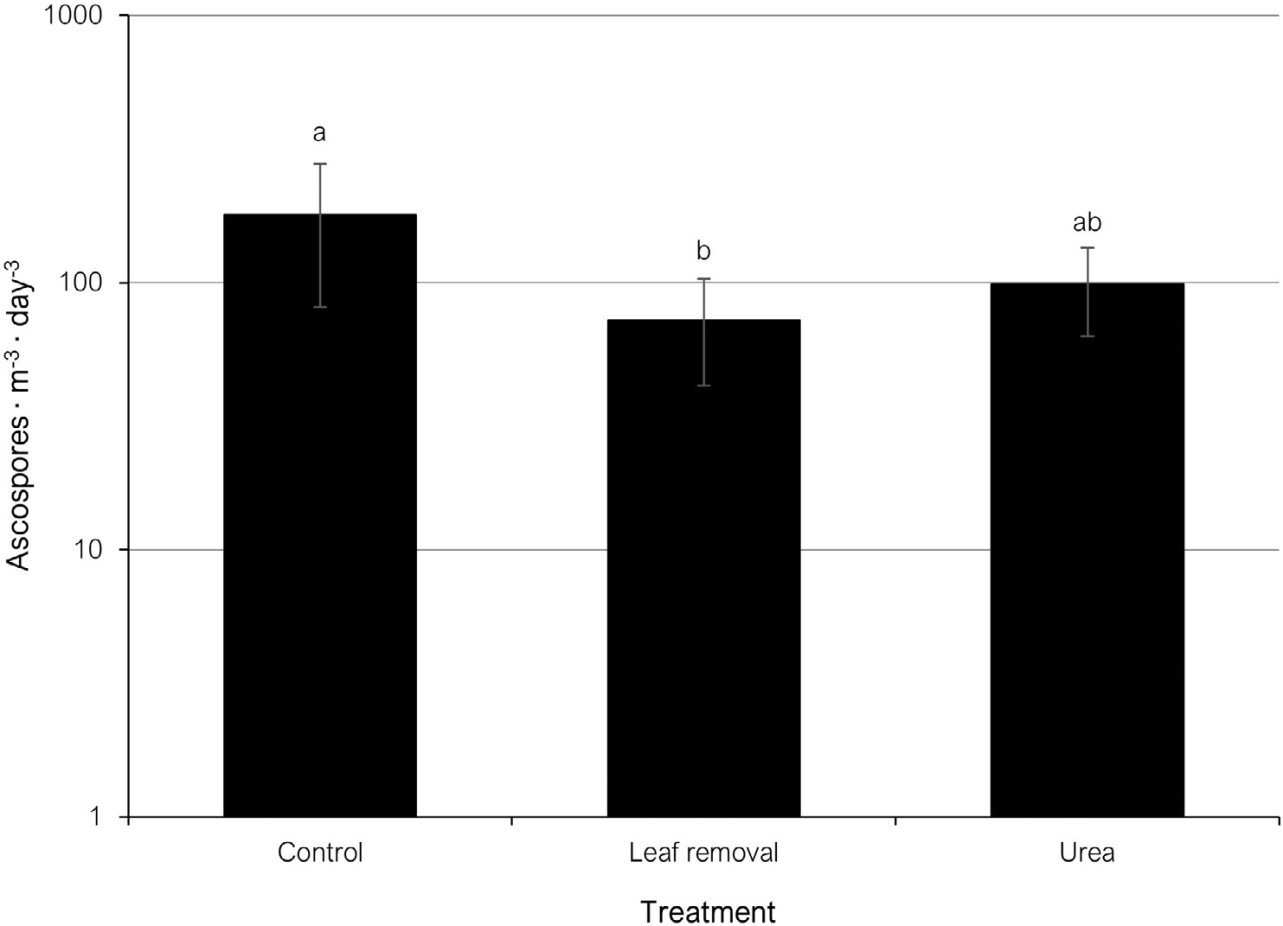
Mean weekly cumulative concentrations of airborne *Polystigma amygdalinum* ascospores in an almond orchard managed with different cultural practices in 2020 and 2022 (combined data). Different letters indicate significant differences according to Tukey–Kramer's test (*P* < 0.05). The error bars indicate the standard error of the mean.

Airborne ascospores of *P. amygdalinum* were detected and quantified in both years for the three evaluated cultural practices. Total accumulated values of daily ascospore concentrations for the untreated control in 2020 were 502.2 asc m^−3^ day^−1^ (range of weekly values: 0.0 to 164.9 asc m^−3^ day^−1^), followed by the urea treatment with 307.0 asc m^−3^ day^−1^ in the same period (range of weekly values: 0.0 to 139.5 asc m^−3^ day^−1^). For the leaf removal treatment, only 1.8 asc m^−3^ day^−1^ were detected in week 15, while the remaining weekly values were zero. In 2022, overall amounts of captured ascospores were higher than in 2020. Thus, total accumulated values of daily ascospore concentrations for the untreated control were 2327.3 asc m^−3^ day^−1^ (range of weekly values: 3.2 to 1571.5 asc m^−3^ day^−1^). For the urea treatment, accumulated total was 1273.5 asc m^−3^ day^−1^ (range of weekly values: 3.9 to 479.0 asc m^−3^ day^−1^). The figures for the leaf removal treatment were comparable to those of the urea treatment, with a total of 1151.0 asc m^−3^ day^−1^ (range of weekly values: 31.8 to 460.9 asc m^−3^ day^−1^). When combining 2020 and 2022 data, mean weekly cumulative concentrations of airborne *P. amygdalinum* ascospores recorded between weeks 15 and 23 were 179.7 asc m^−3^ day^−1^ in the untreated control, 72.1 asc m^−3^ day^−1^ in the leaf removal treatment, and 98.8 asc m^−3^ day^−1^ in the urea treatment. Significant differences were only observed between the control and the leaf removal treatment (Fig. 5).

**Figure 6:**
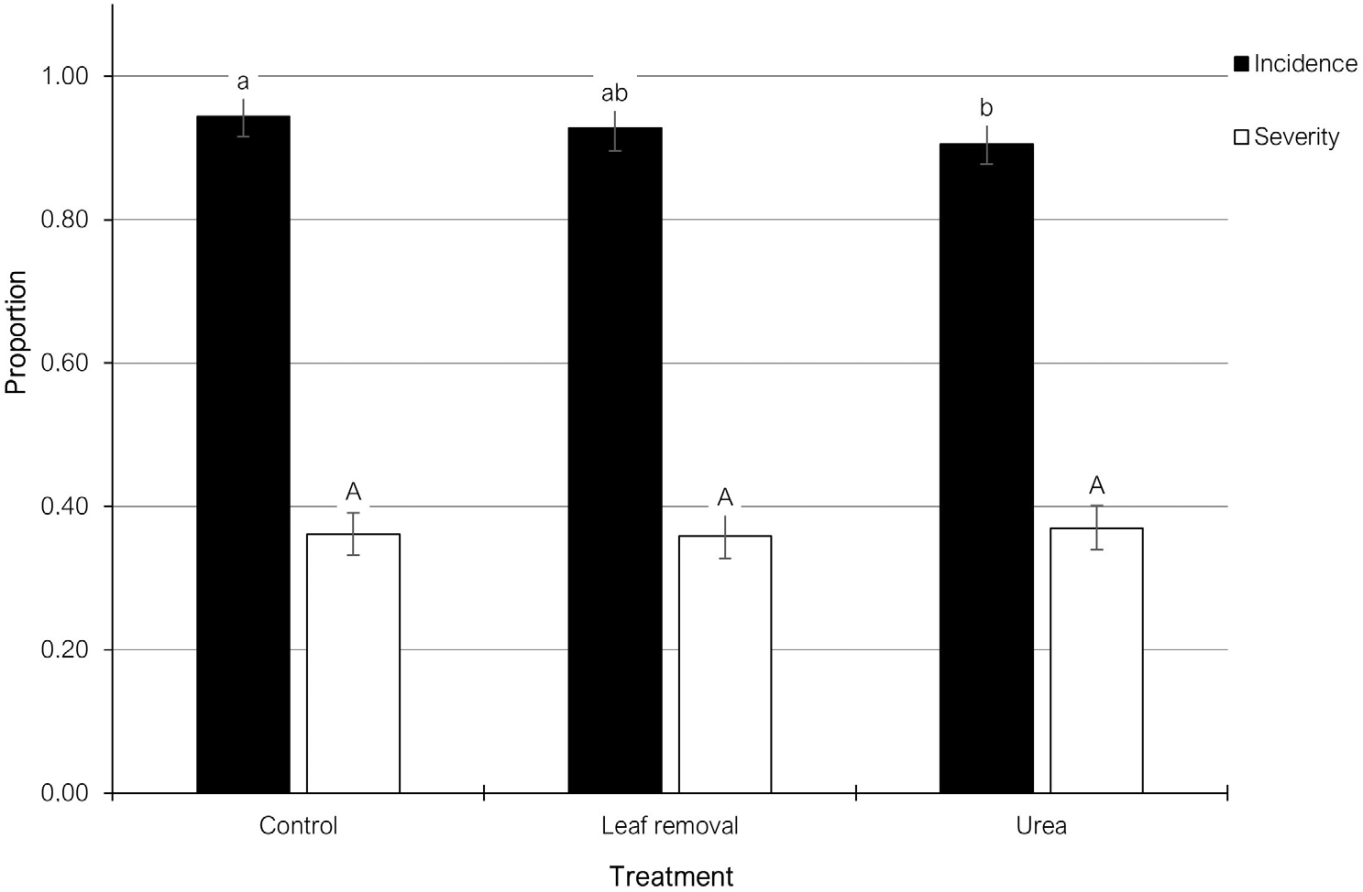
Mean disease incidence and severity of red leaf blotch in almond trees managed with different cultural practices in 2020 and 2022. Different letters indicate significant differences according to Tukey–Kramer's test (*P* < 0.05). The error bars indicate the standard error of the mean.

RLB symptoms were evaluated in 2020 and 2022. In 2020, mean values of RLB incidence ranged between 94.8% (urea treatment) and 99.3% (both leaf removal and the untreated control). RLB severity mean values for the same season ranged between 42.1% (control) and 44.0% (leaf removal). Significant differences were detected in terms of RLB incidence between urea and the other two treatments, whereas no significant differences were detected among treatments for the RLB severity. In 2022, the mean values of RLB incidence ranged between 86.3% (leaf removal and urea treatments) and 89.6% (untreated control), whereas RLB severity ranged from 27.8% (leaf removal) to 31.6% (urea). No significant differences were detected among treatments in any of the dependent variables analyzed. When combining 2020 and 2022 data, mean RLB incidence ranged between 90.6% (urea treatment) and 94.4% (control), RLB severity ranged between 35.9% (leaf removal) and 37.0% (urea). A significant reduction in RLB incidence was observed in the urea treatment (around 0.04 units) as compared to the untreated control. However, no significant differences were observed among treatments concerning RLB severity (Fig. 6).

## 4 DISCUSSION

The successful control of monocyclic diseases such as RLB mainly relies on the reduction of the primary inoculum and the prevention of early infections in spring.^23^, ^24^ In this regard, various cultural practices have been recommended to reduce RLB primary inoculum.^8^, ^22^, ^23^ However, to the best of our knowledge, their direct impact on reducing RLB disease incidence has not been studied. Our study focused on the effects of four chemical products on the reduction of *P. amygdalinum* primary inoculum in almond leaf litter, as well as the effect of the two most recommended cultural practices (urea treatment and leaf litter removal) on the *P. amygdalinum* ascospore release and a further potential reduction in RLB incidence.

Regarding the evaluation of chemicals, the abundance of ascospores in leaf litter was measured three times along the whole RLB infectivity period (weeks 15 to 23) in Catalonia.^13^ It was determined that the optimal data to evaluate the ascospores concentrations is in May, where the highest ascospore amounts were found in the untreated control, and significant differences were additionally found between products. Thus, urea and lime sulfur treatments significantly reduce ascospore production by more than 90%. Lime‐sulfur has been reported to reduce the incidence of blossom blight and brown rot in other *Prunus* crops.^34^ Interestingly, the indirect estimations of *P. amygdalinum* biomass through qPCR demonstrated a consistent fungal biomass reduction in leaf litter treated with urea, in accordance with a recent study performed in Spain which showed that urea was the most effective product for the reduction of RLB primary inoculum.^24^ As for the two cultural practices assessed under field conditions (i.e., urea treatment and leaf litter removal), both treatments showed partial reduction in both airborne ascospores of *P. amygdalinum* and RLB incidence, but differences depended on the treatment and evaluated variable. Thus, only leaf removal resulted in a significant reduction in ascospore trapping, whereas only urea caused a significant decrease in RLB incidence, although by a slight 4%. Nevertheless, the present study is the first to demonstrate a partial reduction in the incidence of RLB through the application of cultural practices in a commercial almond orchard. In other field studies, the degradation effect due to urea has been proven useful for the leaf litter management of apple scab,^35^ and RLB in almond,^24^ but the latter on a small‐scale basis. Similarly, leaf removal has been shown to effectively reduce primary inoculum of apple scab, with significant reductions in both airborne ascospores and subsequent disease incidence and severity.^36^, ^37^

It should be noted that our results indicate that urea could be an effective method to reduce the pressure of RLB primary inoculum. By decreasing the amount of *P. amygdalinum* ascospores and limiting the presence of the fungus in almond leaf litter, urea can help in lowering the weekly cumulative amounts of airborne *P. amygdalinum* ascospores and the overall disease incidence. However, under field conditions, this reduction in ascospores may not be sufficient to significantly decrease the disease severity, as seen in our study. Although the experimental field plots had an average area of 1.6 ha and spore traps were placed roughly at the center of each plot, only minor reductions in airborne spore concentrations were recorded. As previously mentioned, this resulted in the absence of significant differences in disease severity among treatments. This outcome suggests that pathogen colonization and infection may have originated from adjacent fields where the disease was present —as later visually confirmed— likely facilitated by ascospores with a high potential for long‐distance dispersal. Unfortunately, no data are currently available regarding the aerial dispersal dynamics of *P. amygdalinum* ascospores. Therefore, this hypothesis should be addressed in future research. Another important factor is that ascospore concentrations observed in our trial was sufficient overall to trigger RLB symptom development in all treatment plots, resulting in no significant differences in disease severity among the cultural practices studied. This could be attributed to high initial ascospore concentrations originating from adjacent plots or to the possibility that even low ascospore levels are enough to cause symptoms in the field. Should long‐distance dispersal of *P. amygdalinum* ascospores be confirmed, it would underscore the need for implementing cultural control at a regional scale to effectively reduce airborne inoculum pressure. Previous studies on the long‐range dissemination of ascospores^27^, ^38^ further support the rationale for adopting area‐wide disease management strategies for RLB and comparable pathosystems.

Another key difference between the two experiments presented in this study lies in the urea dosage used. In the preliminary screening of chemical products, a higher concentration of urea (100 g L^−1^) markedly reduced ascospore production. In contrast, a lower concentration (25 g L^−1^) was used in the field trial, in accordance with label recommendations, which did not result in comparable results. These findings suggest that higher urea concentrations may be required to achieve effective ascospore control under field conditions. However, application rates must comply with regulatory guidelines.

The results of the current research provide valuable insights to manage the primary inoculum of *P. amygdalinum* in commercial almond orchards. However, it is important to note the complexity of controlling RLB under field conditions using cultural practices alone. Therefore, a long‐term strategy combining all available practices would be required to significantly reduce RLB infections. This strategy should include: (i) the use of tolerant varieties, (ii) the application of cultural practices to reduce the primary inoculum in leaf litter, and (iii) the use of preventive fungicide applications in combination with the use of forecasting epidemiological models.

## 5 CONCLUSIONS

Our findings may contribute to essential information on almond leaf litter management that can help in growing healthier almond orchards. Cultural practices, such as the application of urea on the almond leaf litter or, alternatively, its removal, can help in the control of the primary inoculum of *Polystigma amygdalinum*. However, to enhance an overall sustainable management of almond red leaf blotch, these techniques should be integrated into a broader strategy that should include the use of preventive fungicides associated with forecasting models, and the use of tolerant cultivars.

## AUTHOR CONTRIBUTIONS

Conceptualization, L.T., J.L. and X.M.; methodology, all authors; validation, J.L. and X.M.; formal analysis, J.L. and G.P‐S.; investigation, G.P‐S., J.L., L.T. and X.M.; resources, J.L., L.T. and X.M.; data curation, G.P‐S. and J.L.; writing—original draft preparation, G.P‐S., J.L. and L.A.‐D.; writing—review and editing, all authors; visualization, G.P‐S. and L.A.‐D.; supervision, J.L., L.T. and X.M.; project administration and funding acquisition, J.L., L.T. and X.M. All authors have read and agreed to the published version of the manuscript.

## FUNDING INFORMATION

Research funded by the Instituto Nacional de Investigación y Tecnología Agraria y Alimentaria (INIA, Spain), with grant RTA2017‐00009‐C04‐01, and Agencia Estatal de Investigación (AEI, Spain) with grant PID2020‐114648RR‐C31 (MCIN/AEI/10.13039/501100011033). All authors, except G.P.‐S., were supported by the CERCA Program, Generalitat de Catalunya. G.P.‐S. was supported by the Agencia Estatal de Investigación (AEI), Spain, through a predoctoral grant (PRE2018‐085207).

## CONFLICTS OF INTEREST

The authors declare no commercial or financial relationships that could be construed as a potential conflict of interest. Commercial products mentioned in this article were used solely for methodological purposes and their mention does not imply endorsement or recommendation by the authors.

## Supporting information


**Data S1:** Supporting Information.

## Data Availability

The data that support the findings of this study are openly available in CORA (Catalan Open Research Area. Repositori de Dades de Recerca) at https://doi.org/10.34810/data2346.
