# Supplementary material for: Assessing alternative strategies to control almond red leaf blotch through the reduction of Polystigma amygdalinum inoculum in leaf litter
Source: Pest Manag Sci. 2025 Sep 8;82(1):278–86. doi: 10.1002/ps.70192 (PMC12713699; doi:10.1002/ps.70192)
Supplement: Supplementary file 1 — Data S1: Supporting Information. [file PS-82-278-s001.docx]

**Supplementary material**

**Figure S1**. Mean amounts of *Polystigma amygdalinum* ascospores in almond leaf litter, expressed as ascospores per gram of dried leaf weight, treated with different chemical products and collected in May of 2022 and 2023 seasons. Different letters indicate significant differences according to Tukey-Kramer’s test (*P* < 0.05). The error bars indicate the standard error of the mean.

**Supplementary material**

**Figure S2**. Mean quantification cycle values (Cq) from qPCR analyses targeting *Polystigma amgydalinum* in almond leaf litter treated with different chemical products and collected in May of 2022 and 2023 seasons. Different letters indicate significant differences according to Tukey-Kramer’s test (*P* < 0.05). The error bars indicate the standard error of the mean.
